# Centrifugation versus PureGraft for fatgrafting to the breast after breast-conserving therapy

**DOI:** 10.1186/1477-7819-12-178

**Published:** 2014-06-05

**Authors:** Ondrej Mestak, Andrej Sukop, Yu-Sheng Hsueh, Martin Molitor, Jan Mestak, Jana Matejovska, Lucie Zarubova

**Affiliations:** 1 Department of Plastic Surgery, Bulovka Hospital, 1st Medical Faculty of Charles University in Prague, Budinova 2, Prague 8 180 00, Czech Republic; 2 Department of Plastic Surgery, 3rd Medical Faculty of Charles University in Prague, Prague, Czech Republic; 3 Institute of Biomedical Engineering, College of Medicine and College of Engineering, National Taiwan University, Taipei 100, Taiwan

**Keywords:** Fatgrafting, Breast-conserving treatment, Breast reconstruction

## Abstract

**Background:**

Breast-conserving treatment (BCT) leads to a progressive and deteriorating breast deformity. Fatgrafting is ideal for breast reconstruction after BCT. The most frequently utilized technique for fat processing is centrifugation. The PureGraft device (Cytori Therapeutics, San Diego, CA, USA) is a new method that involves washing and filtering the fat to prepare the graft. We compared the subjective and objective outcomes of two fat-processing methods, centrifugation and PureGraft filtration.

**Methods:**

Thirty patients underwent breast reconstruction performed by a single surgeon (OM) after BCT in our department between April 2011 and September 2012. The patients were preoperatively divided into two groups randomly: 15 received fatgrafts processed by centrifugation, and 15 received fatgrafts processed by washing in PureGraft bags. The patients were followed up for 12 to 30 months. To measure the subjective outcome, we distributed the BREAST-Q questionnaire to all the patients both preoperatively and 1 year postoperatively. The BCCT.core software evaluated the objective outcome of breast reconstruction by fatgrafting.

**Results:**

The Breast-Q results indicated a tremendous improvement in the modules “Satisfaction with Breast” and “Psychosocial Well-being”. The “Sexual Well-being” scale also improved. Only the module “Satisfaction with Breasts” significantly differed between groups; patients treated with the PureGraft fat exhibited better outcomes. The BCCT.core results did not significantly differ between the groups.

**Conclusion:**

One year postoperatively, the outcomes of the use of PureGraft bags or centrifugation to process fat for breast reconstruction after BCT did not differ. The unpredictability of the results following fatgrafting procedures is likely due to interindividual differences with yet-undisclosed causes.

## Background

In conjunction with improvements in non-surgical techniques for treating breast cancer over the last decade, surgical treatment has also moved toward a more conservative approach.

In early-stage breast cancer, breast-conserving treatment (BCT), consisting of a lumpectomy followed by radiation, is considered the standard of care [1-3]. Due to improved screening, earlier treatment and modern treatment protocols, the number of women surviving breast cancer is increasing. Although these patients are cured of their disease, the treatment can be followed by unwanted consequences including breast deformity. Modern treatment paradigms are evolving, with the goal of improved cancer-related outcomes and reduced late effects. Body image and quality of life are improved in women following BCT compared with mastectomy [4]. Nevertheless, BCT leads to breast deformity in most patients [5-7]. As a result of breast irradiation, the deformity is progressive and deteriorates with time.

Although breast reconstruction after a modified radical mastectomy is straightforward (many reconstructive techniques are available), breast reconstruction after BCT has been problematic. Two factors must be considered. The first is the uneven breast shape resulting from partial mastectomy. The second is the pre-existing irradiation of the breast. A silicone implant does not correct the uneven breast shape and is not the best option for the reconstruction of irradiated tissue [8]. Among other techniques used for breast reconstruction after BCT are latissimus dorsi flap, thoracodorsal artery perforator flap and intercostal artery perforator flap.

Fatgrafting (autologous fat transfer) has experienced a tremendous boom in recent years. There had been a negative attitude toward this method, including suspicions that it caused necrosis and colliquation and that the whole graft may be quickly absorbed [9]. However, since 1990, an increasing number of plastic surgeons have begun to add this method, following improvements in the techniques and extensive experimental and clinical studies, to their armamentarium [10-12]. Fat is a filler with ideal properties; for example, it naturally integrates into tissues, is autologous and is 100% biocompatible.

The filler function, however, is not the most important aspect of using autologous fat tissue. An increasing number of published studies have reported the regenerative effects of autologous fatgrafts [13,14]. These remarkable regenerative effects are particularly observed in irradiated areas. The regenerative effect is most likely due to the high content of mesenchymal stem cells in the fatgrafts (approximately 300,000/cm^3^ of fat tissue) [15].

**Figure 1:**
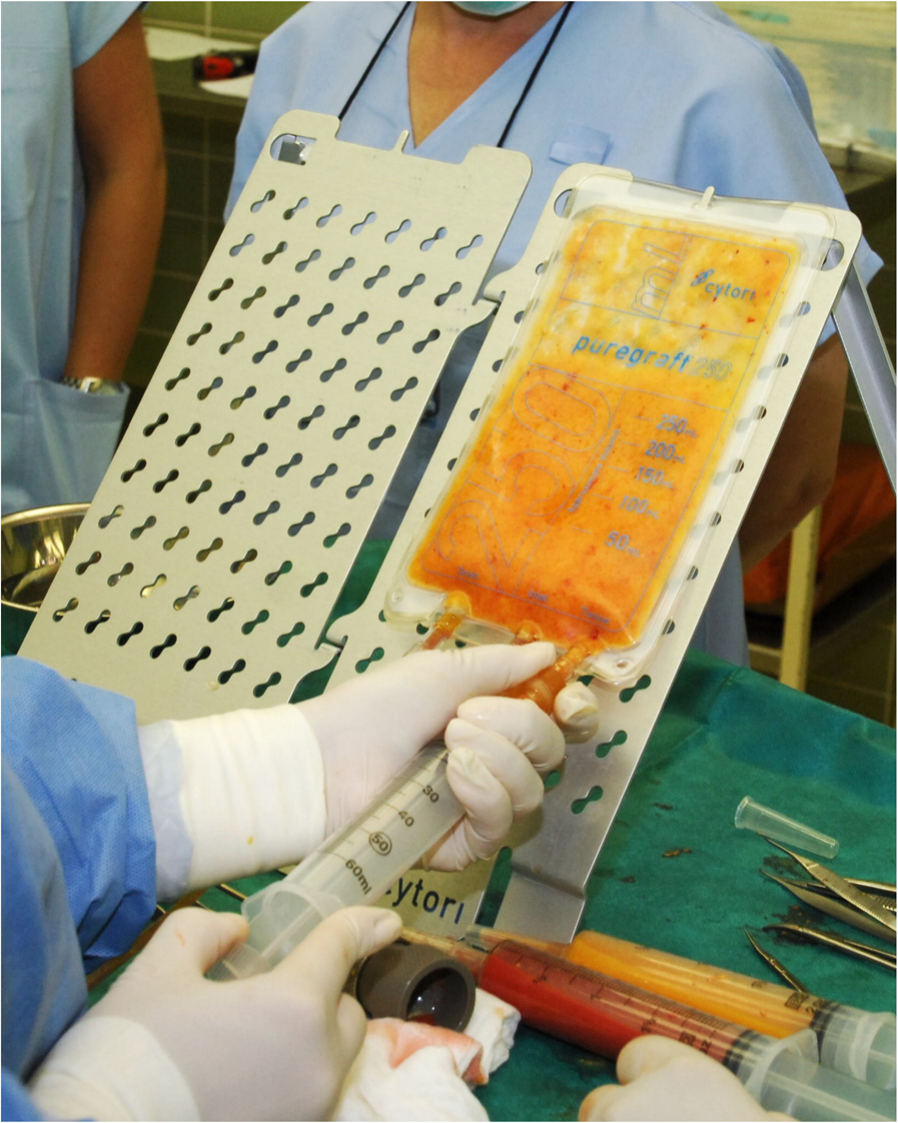
Aspirating processed fatgraft from the PureGraft (Cytori Therapeutics, San Diego, CA, USA).

In today’s clinical practice, several fat-processing techniques are available. These processing methods are particularly aimed at increasing the predictability of fatgrafting, which can benefit substantially from improvements. The most commonly used technique for fat processing is centrifugation, which is typically known as part of the “Coleman technique” (fat centrifugation in 10 cm^3^ syringes, for 3 minutes at 3,000 rpm) [14]. Some of the new techniques offer much faster graft processing, which is beneficial, particularly for large-volume fatgrafting. One such method employs the PureGraft device (Cytori Therapeutics, San Diego, CA, USA), which involves washing and filtering fat to prepare the grafts (Figure 1). However, cytokines important for tissue regeneration and graft survival [16] may be washed away during processing with this method.

We hypothesized that using PureGraft bags for processing fat could lead to a decreased regenerative effect, quicker absorption of the fatgraft and less predictable results. Many studies have evaluated fatgraft composition, using different harvesting and processing methods [17-19]. Even so, few clinical studies have sufficiently evaluated the outcomes of different processing methods [20-22]. Therefore, the aim of our study was to compare the subjective and objective outcomes of two fat-processing methods: centrifugation and filtration in a PureGraft device. We chose two primary methods for outcome assessment, including a patient-reported outcome questionnaire (Breast-Q) and a computer system (BCCT.core) to objectively evaluate the results.

## Methods

Patients who underwent breast reconstruction after BCT performed by a single surgeon (OM) in our department between April 2011 and September 2012 were preoperatively and randomly divided into two groups. The first group received fatgrafts processed by filtration in a PureGraft device, and the second group received fatgrafts processed by centrifugation.

The protocol was approved by the ethical review board of Bulovka Hospital in Prague, and all patients signed informed consent before participating in the study.

All procedures were performed under general anesthesia. Before liposuction, we infiltrated the subcutaneous tissue with a tumescent solution containing 1 l of saline and 1 ml of adrenaline. Fat was harvested from the abdominal wall, the flanks or the lateral thighs using a 60 ml Toomey syringe (handheld) and a 3 mm Mercedes cannula (Mentor, Santa Barbara, CA, USA). In the first group, we processed the fat in 250 cm^3^ PureGraft bags (Cytori Therapeutics), using two washes with 150 cm^3^ Ringer’s solution. In the second group, the fat was processed by a 3-minute, 3,000 rpm centrifugation in 10 cm^3^ cannulas in a centrifuge (Medilite, Mentor, Santa Barbara, CA, USA). The graft was injected into many sites around the reconstructed area, using a 9 cm type III Coleman cannula (Mentor). The injection was performed very slowly with a fan-shaped technique during the withdrawal of the cannula. We attempted (as much as possible) to prevent accumulation of the fatgraft and overfilling of the tissue to prevent ischemia, necrosis, colliquation and calcification. We perform mild rigottomies (releasing of scar with needle) in virtually all procedures.

The patients were administered antibiotics prophylactically before the operation. The patients wore elastic banding only on the liposuctioned areas, and the breasts were left without pressure. The patients were discharged from the hospital 1 day postoperatively.

To measure subjective outcomes, we used the BREAST-Q questionnaire (Memorial Sloan-Kettering Cancer Center and The University of British Columbia), which we distributed to all patients preoperatively and 1 year postoperatively. The BREAST-Q is a patient-reported outcome instrument [23-26]. It is comprised of the following two overarching domains: Patient Satisfaction and Health-related Quality of Life. The Quality of Life domain contains several modules; the “Psychosocial Well-being” module measures psychosocial well-being with items that ask about body image (for example, acceptance of one’s body; attractiveness) and a woman’s confidence in social settings. Other items cover emotional health and self-esteem. The “Sexual Well-being” module measures sexual well-being and body-image issues with items that ask about feelings of sexual attractiveness when clothed and unclothed and sexual confidence as it relates to one’s breasts, as well as how comfortable or at ease a woman feels during sexual activity. The “Physical Well-being” module measures physical problems such as pain (for example, neck, back, shoulder, arm, rib) and problems in the breast area (for example, tightness, pulling, tenderness, pain).

The Satisfaction domain primarily contains the “Satisfaction with Breasts” module*,* which measures body image in terms of a woman’s satisfaction with her breasts and asks questions regarding how comfortably bras fit and how satisfied a woman is with her breast area, both clothed and unclothed. Postoperative items ask about breast appearance (for example, size, symmetry, softness) and clothing issues (for example, how bras fit, being able to wear fitted clothes).

The patients’ responses to each scale’s items are transformed through the Q-Score scoring software to provide a total scale score that ranges from 0 to 100. For all BREAST-Q scales, a higher score indicates greater satisfaction or better quality of life. A mean change of 5 to 10 on a multi-item scale is perceived as ‘a little’ change, 10 to 20 as ‘a moderate’ change and greater than 20 as ‘a maximal’ change.

The objective outcome of breast reconstruction by fatgrafting was evaluated using BCCT.core software [27], which was developed by The University of Porto to evaluate the cosmetic results of BCT in a semiautomatic, objective manner. Standardized, digital, front photographs of patients were taken preoperatively and 1 year postoperatively. In the BCCT.core software, we manually marked the position of an infra-mammary line and nipple. The system divided the results into four categories (excellent = 1, good = 2, fair = 3 and bad = 4).

In addition, we evaluated local findings on the breast, including changes of the texture and palpable lumps post-fatgrafting (small and large); postoperative radiologic examinations; and operative time.

The data were analyzed statistically as follows: quantitative data using the Wilcoxon test and categorical data using the nonparametric Pearson chi-square, Mann–Whitney and Fisher tests.

## Results

### Surgeries

**Figure 2:**
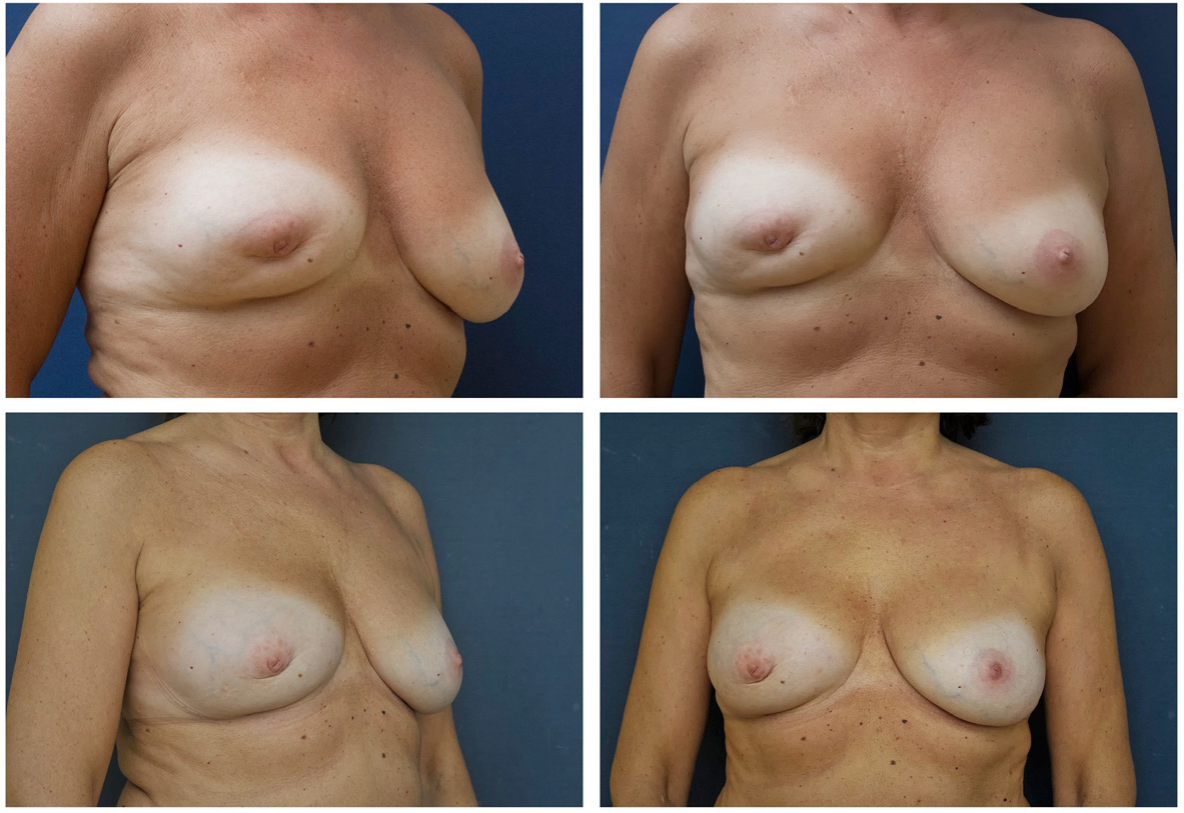
Representative patient before (top) and 12 months after (below) breast reconstruction after breast conserving therapy using a fatgraft processed by centrifugation.

**Figure 3:**
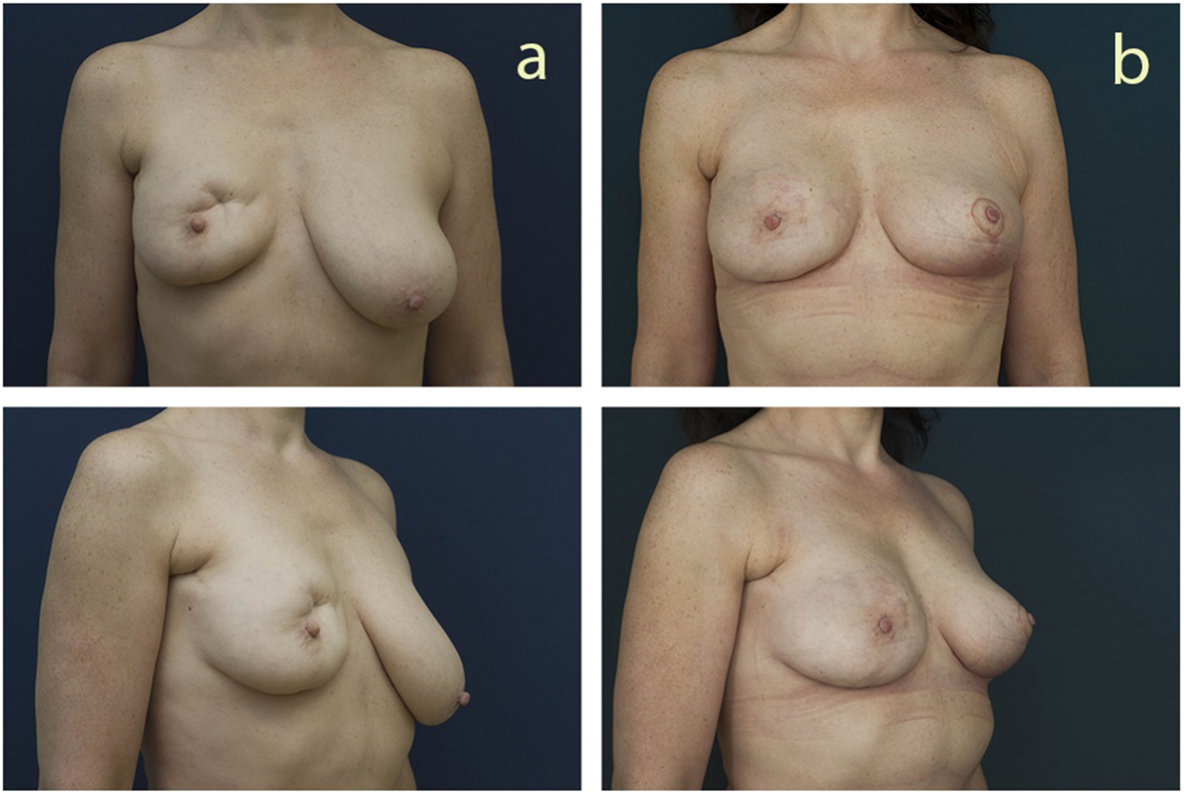
Representative patient before (a) and 12 months after (b) breast reconstruction after breast conserving therapy using a fatgraft processed by the PureGraft (Cytori Therapeutics, San Diego, CA, USA) method.

Between April 2011 and September 2012, a single surgeon (OM) operated on 30 patients. Their mean age was 38.3 years (28 to 62 years). Fifteen patients received a fatgraft processed by centrifugation (20.6 (ranged from 9 to 37) months from BCT) (Figure 2); 15 patients received a fatgraft processed by washing in PureGraft bags (23.1 (ranged from 8 to 48) months after BCT) (Figure 3). The patients were followed up for 12 to 30 months (mean 21 months). Patients in the centrifugation group underwent surgery 10 to 46 (mean 24.6) months after BCT treatment, and patients in the PureGraft group underwent surgery 1 to 28 (mean 20.5) months after BCT treatment. We injected 80 to 300 cm^3^ (mean 162 cm^3^) of the graft in the centrifugation group and 80 to 340 cm^3^ (mean 232 cm^3^) in the PureGraft group.

A surgical-site infection occurred in one patient from the PureGraft group in one breast area. She was treated with antibiotic therapy and drainage. The infection resulted in partial loss of the graft. Upon ultrasonography, solitary cysts (smaller than 1 cm) were observed in two patients from the PureGraft group and in one patient from the centrifuge group. In both groups, there were no other complications, such as cysts, fat necrosis, palpable lumps or ultrasonographic abnormalities, during the postoperative course.

### Breast Q

**Figure 4:**
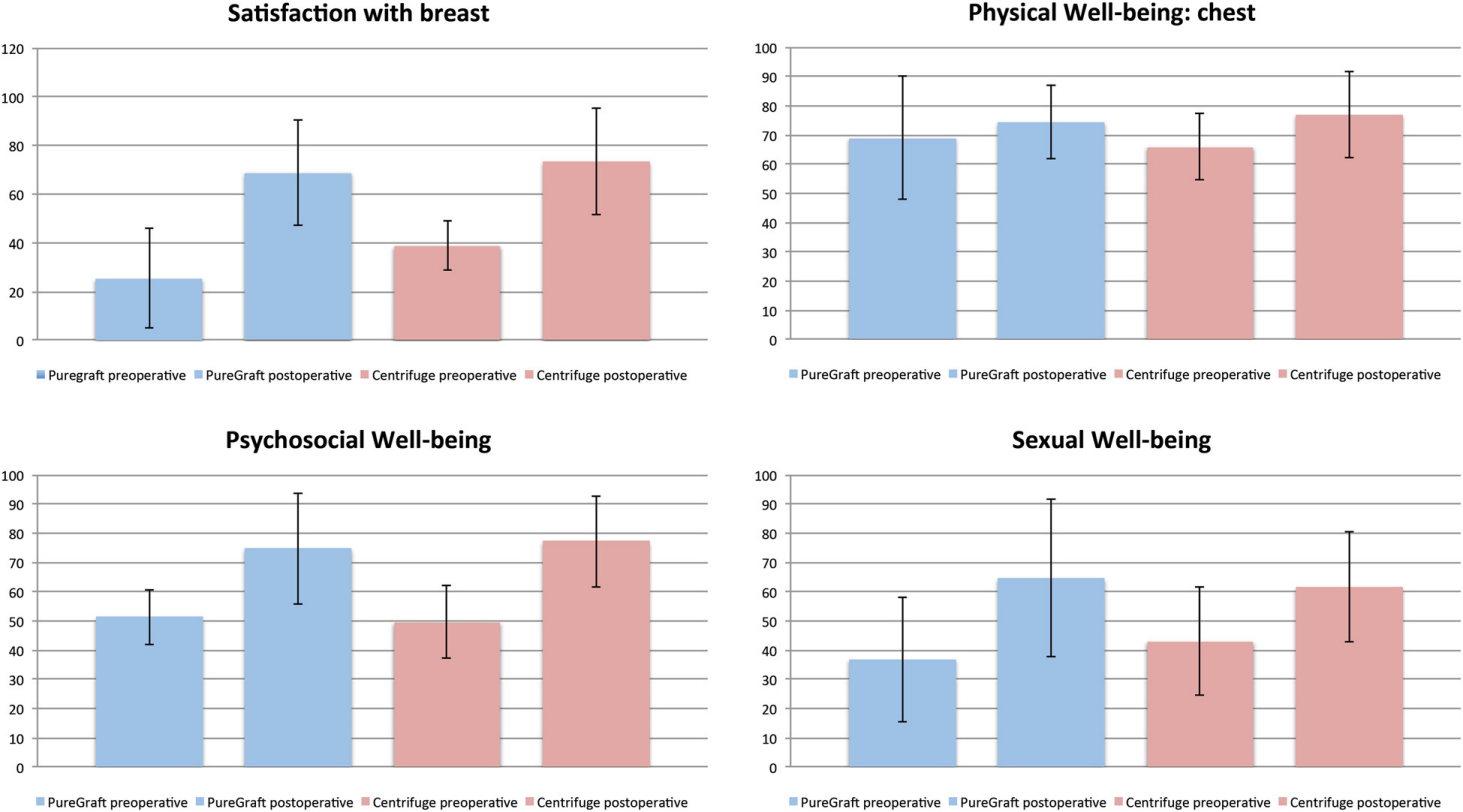
**Results evaluated by the breast-Q instrument.** We found a tremendous improvement in the “Satisfaction with Breast” and “Psychosocial Well-being” modules in both groups. The only module in which we did not observe an enhancement was “Physical Well-being: chest”.

We analyzed the patient-measured outcomes in five modules, as follows (Table 1, Figure 4):

**Table 1:** Values of the results evaluated by the breast-Q instrument

	**Module**			
	**Satisfaction with breast**	**Psychosocial well-being**	**Physical well-being: chest**	**Sexual well-being**
PureGraft preoperative	25.3	51.3	69	36.8
PureGraft postoperative	68.8	74.7	74.4	64.5
Change	+52.6	+32.8	+2	+34.8
Centrifuge preoperative	38.8 (*P* = 0.75)	49.6	66	43
		(*P* = 0.638)	(*P* = 0.671)	(*P* = 0.538)
Centrifuge postoperative	73.5 (*P* = 0.697)	77.2	77	61.5
		(*P* = 0.562)	(*P* = 0.605)	(*P* = 0.847)
Change	+38.4 (*P* = 0.568)	+29.3 (*P* = 0.784)	+11.7 (*P* = 0.328)	+20 (*P* = 0.465)

*P* values indicate the significance of comparisons between the centrifugation and PureGraft groups.

“Satisfaction with Breast”: In the PureGraft group, the average scores were 25.3 preoperatively and 68.8 postoperatively (a change of 52.6 - maximal change). In the centrifugation group, the average scores were 38.8 preoperatively and 73.5 postoperatively (a change of 38.4 - maximal change). The observed change did not significantly differ between the two methods (52.6 vs 38.4; *P* = 0.568) (the difference was counted as average of substractions of individual cases).

“Psychosocial Well-being”: In the PureGraft group, the average scores were 51.3 preoperatively and 74.7 postoperatively (a change of 32.8 - maximal change). In the centrifugation group, the average scores were 49.6 preoperatively and 77.2 postoperatively (a change of 29.3 - maximal change). The observed change did not significantly differ between the two methods (32.8 vs 29.3; *P* = 0.784).

“Physical Well-being: Chest”: In the PureGraft group, the average scores were 69 preoperatively and 74.4 postoperatively (a change of 2 - no change). In the centrifugation group, the average scores were 66 preoperatively and 77 postoperatively (a change of 11.7 - moderate change). The observed change did not significantly differ between the two methods (2 vs 11.7; *P* = 0.328).

“Sexual Well-being”: In the PureGraft group, the average scores were 36.8 preoperatively and 64.5 postoperatively (a change of 34.8 - maximal change). In the centrifugation group, the average scores were 43 preoperatively and 61.5 postoperatively (a change of 20.1 - maximal change). The observed change did not significantly differ between the two methods (34.8 vs 20.1; *P* = 0.465).

**Figure 5:**
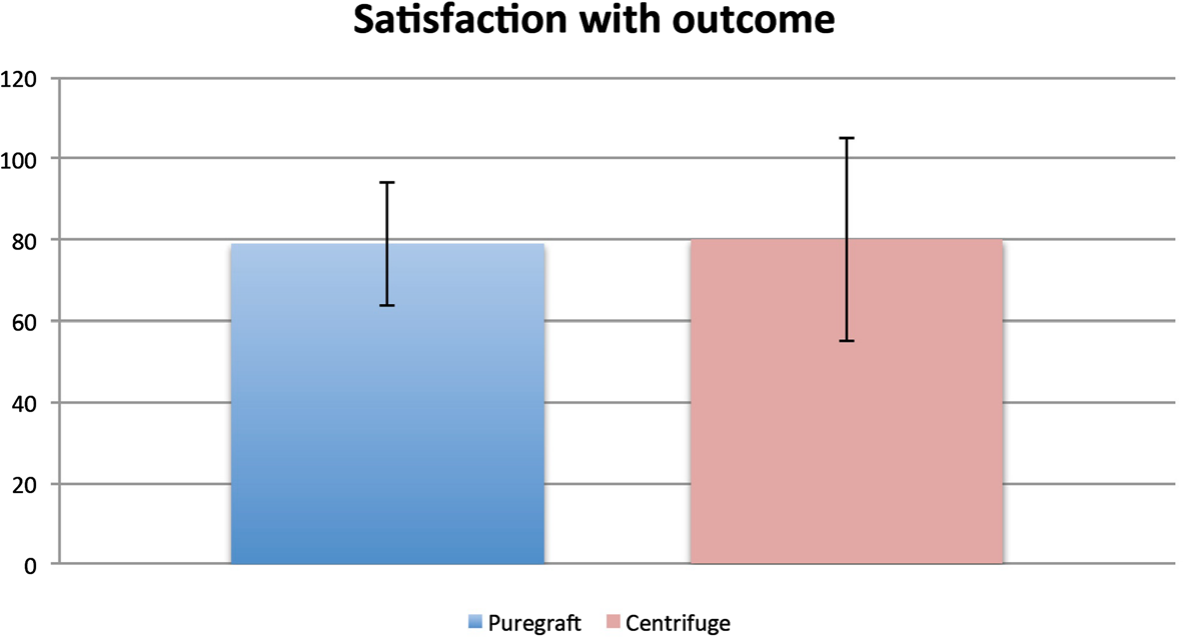
The “Satisfaction with outcome” module of breast-Q instrument was administered only postoperatively, and the difference between groups was statistically insignificant.

The “Satisfaction with Outcome” questionnaire was administered only postoperatively. The results were 79 points for the PureGraft group and 80 points for the centrifuge group (*P* = 0.643) (Figure 5).

### BCCT.core software

**Figure 6:**
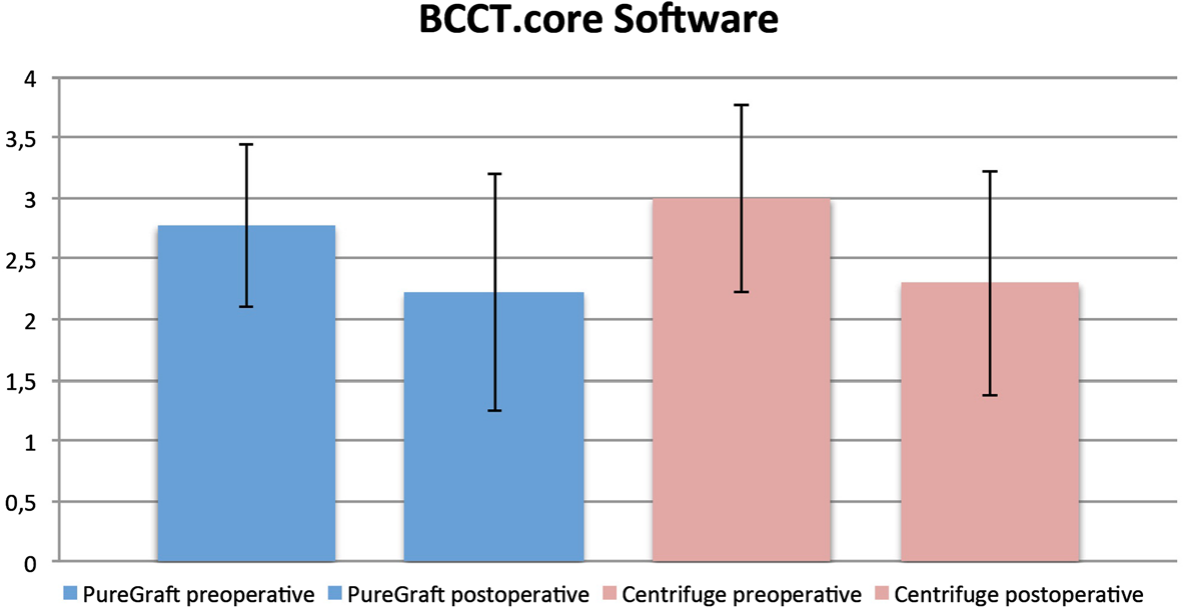
**Results evaluated by the BCCT.core software*.*** A comparison of the two groups indicated that the difference was statistically insignificant.

In the PureGraft group, there was a change from 2.77 points before the operation to 2.22 points after the operation (an improvement of 0.55 points). In the centrifugation group, the average score changed from 3 preoperatively to 2.3 postoperatively (an improvement of 0.63 points) (Figure 6). A comparison of the two groups (0.63 vs 0.55) indicated that the difference was not statistically significant (*P* = 0.859).

## Discussion

Considering its flexibility, excellent postoperative results and low incidence of complications, fatgrafting is appreciated by patients after breast cancer therapy. In recent years, this method has changed the outlook on breast operations that include plastic surgery. However, plastic surgeons are in a never-ending search for ideal techniques for fat harvesting, processing and injection. A tremendous selection of techniques is available, particularly for fat processing, including centrifuges with different speeds, diameters, volumes and tube shape capacities. In addition to centrifuges, however, there are other available techniques for fat processing, such as filtering over a simple mesh or using more sophisticated devices such as the PureGraft. The PureGraft has several practical advantages over centrifugation. First, it is a closed system, which prevents graft infection (unlike centrifugation in the open air). The second advantage of the PureGraft over the centrifuge is the speed of processing; it requires approximately 12 minutes to obtain 300 cm^3^ of a graft ready for injection using the PureGraft device, whereas it requires almost twice as long when processed by centrifugation. The drawback of the PureGraft device is that it can eliminate important substances (such as cytokines) that are needed for graft survival and the regeneration effect. Therefore, we decided to perform a clinical study on a group of patients undergoing breast reconstruction after BCT. Several studies have evaluated different fatgraft-processing methods, but few have had valid outcomes. In the present study, we utilized two methods for the outcome assessment: a patient-reported outcome questionnaire (Breast-Q) and a computer system (BCCT.core) to objectively evaluate the results.

The importance of patient-reported outcomes is becoming increasingly well recognized in the medical community. Most published papers discuss postoperative morbidity only in terms of evaluating locoregional recurrence. Cosmetic results are often insufficiently evaluated by a group of physicians, retrospectively from physician documentation [28,29] or by simple non-standardized questionnaires that rate the cosmetic results as excellent, good, fair or poor [30].

When evaluating the Breast-Q results, we found a tremendous improvement in the “Satisfaction with Breast” and “Psychosocial Well-being” modules. There was also improvement in the “Sexual Well-being” scale, and the “Satisfaction with Outcome” scores were high. The only module in which we did not observe an enhancement was “Physical Well-being: Chest”, which we found surprising. We expected that there would be more improvement in this scale due to the regenerative effect on the irradiated tissue. Our unexpected result might have been caused by the structure of the questions in this module, which also addressed symptoms such as back pain.

A computer system (BCCT.core) has been developed to evaluate the aesthetic results of BCT objectively and automatically, and this system is a standard method for assessing the cosmetic outcomes of BCT [27]. In both groups, only a slight change occurred in the BCCT.core score (0.55 for the PureGraft group vs 0.63 for the centrifugation group). In our opinion, this result was caused by the marked breast asymmetry that followed radiotherapy. We treated the defects after lumpectomy; in some cases, we performed augmentation of the whole breast. Most of the patients had a contralateral ptotic breast, which appeared to the software as significant asymmetry and was not corrected by fatgrafting.

Every patient was regularly followed-up by the oncologist after this procedure. Fatgrafting to the breast after BCT is consistent with oncological safety. Experimental [31] and clinical [32,33] studies do not show increased risk of locoregional relapse of breast cancer. Petit and colleagues [32] described a series of 646 patients who had undergone fatgrafting after breast cancer therapy. They were followed up for a mean of 19.2 months and the locoregional relapse was 2.4%. This number is not significantly different from the risk of locoregional relapse in the group without lipofilling.

There are few clinical reports in the literature that have directly compared fat-processing methods. The results of our study correspond with those of Botti and colleagues [20], who did not find any difference in outcome between wash- or centrifugation-processed fatgrafts. They used grafts for facial fat transplantation in 25 patients. One side of the face was treated using fat processed by a strainer, and the second side was treated with centrifuged fat. Botti and colleagues only evaluated plain photographs in their analysis.

In our opinion, the unpredictability of the results following fatgrafting procedures is due to interindividual differences with yet-undisclosed causes. The processing method is likely insignificant.

## Conclusions

One year postoperatively, the difference between the use of PureGraft bags and centrifugation for fat processing for breast reconstruction after BCT was insignificant. The advantages of the PureGraft device include its closed nature, which prevents graft contamination, and its faster speed for larger volume graft processing.

## Abbreviations

BCT: breast-conserving therapy.

## Competing interests

The authors declare that they have no conflict of interest.

## Authors’ contributions

OM as the principal investigator, participated in data collection, the interpretation of the results, and the writing of the manuscript. AS participated in the interpretation of the results and provided supervision. YH participated in data collection as a clinical collaborator. MM participated in data collection as a clinical collaborator. JM participated in the interpretation of the results and provided supervision. JM participated in data collection as a clinical collaborator. LZ participated in data collection as a clinical collaborator. All authors read and approved the final manuscript.
